# Noninvasive Pressure-Volume Loops Predict Major Adverse Cardiac Events in Heart Failure With Reduced Ejection Fraction

**DOI:** 10.1016/j.jacadv.2024.100946

**Published:** 2024-05-04

**Authors:** Per M. Arvidsson, Jonathan Berg, Marcus Carlsson, Håkan Arheden

**Affiliations:** Department of Clinical Sciences Lund, Clinical Physiology, Skåne University Hospital, Lund University, Lund, Sweden

**Keywords:** cardiovascular magnetic resonance imaging, pressure-volume loop analysis, noninvasive, heart failure, outcome

## Abstract

**Background:**

Heart failure with reduced ejection fraction (HFrEF) is characterized by ventricular remodeling and impaired myocardial energetics. Left ventricular pressure-volume (PV) loop analysis can be performed noninvasively using cardiovascular magnetic resonance (CMR) imaging to assess cardiac thermodynamic efficiency.

**Objectives:**

The aim of the study was to investigate whether noninvasive PV loop parameters, derived from CMR, could predict major adverse cardiac events (MACE) in HFrEF patients.

**Methods:**

PV loop parameters (stroke work, ventricular efficiency, external power, contractility, and energy per ejected volume) were computed from CMR cine images and brachial blood pressure. The primary end point was MACE (cardiovascular death, heart failure (HF) hospitalization, myocardial infarction, revascularization, ventricular tachycardia/fibrillation, heart transplantation, or left ventricular assist device implantation within 5 years). Associations between PV loop parameters and MACE were evaluated using multivariable Cox regression.

**Results:**

One hundred and sixty-four HFrEF patients (left ventricular ejection fraction ≤40%, age 63 [IQR: 55-70] years, 79% male) who underwent clinical CMR examination between 2004 and 2014 were included. Eighty-eight patients (54%) experienced at least one MACE after an average of 2.8 years. Unadjusted models demonstrated a significant association between MACE and all PV loop parameters (*P* < 0.05 for all), HF etiology (*P* < 0.001), left ventricular ejection fraction (*P* = 0.003), global longitudinal strain (*P* < 0.001), and N-terminal prohormone of brain natriuretic peptide level (*P* = 0.001). In the multivariable Cox regression analysis adjusted for age, sex, hypertension, diabetes, and HF etiology, ventricular efficiency was associated with MACE (HR: 1.04 (95% CI: 1.01-1.08) per-% decrease, *P* = 0.01).

**Conclusions:**

Ventricular efficiency, derived from noninvasive PV loop analysis from standard CMR scans, is associated with MACE in patients with HFrEF.

Heart failure with reduced ejection fraction (HFrEF) is defined by impaired systolic function and typically presents with ventricular dilatation, both heralds of myocardial energy waste. Untreated, HFrEF progresses in a ‘downward spiral’ of forward failure, organ hypoperfusion, reflexive renin-angiotensin-aldosterone system stimulation, fluid accumulation, increased preload, further decline in systolic function, and so on. This progressive decline reflects the body’s inherent tendency to prioritize cardiac output and hence cerebral perfusion over long-term cardiac health.

The clinical outcome for HFrEF patients depends largely upon whether the therapeutic strategy successfully targets the specific pathophysiological mechanisms causing forward failure in the individual patient. Regardless of the underlying etiology, however, mitochondrial dysfunction and hence myocardial energetic deficit are near-universal findings in HFrEF,^1^ indicating the central role of the myocardial energy metabolism in the progression of HF. The dramatic improvements in long-term morbidity and mortality seen in the wake of the modern paradigm for medical HF treatment largely arise from augmented myocardial energetics secondary to reduced cardiac loading.^2^^,^^3^ Unloading the heart—improving its chronic working conditions—is key to managing HFrEF.

While risk in HFrEF can be assessed using several established imaging measures of left ventricular (LV) function, including the load-dependent parameters left ventricular ejection fraction,^4^ longitudinal strain,^5^^,^^6^ and atrioventricular plane displacement,^7^ the prognostic value of these methods likely reflects their intrinsic but imperfect relationship to how efficiently the heart functions as a pump. Assessing cardiac thermodynamic efficiency^8^^,^^9^ more directly through pressure-volume (PV) loop analysis^10^^,^^11^ has potential added benefit in HF, as it provides unique windows into physical quantities such as ventricular stroke work (SW), efficiency, external power, and myocardial O_2_ consumption. While current HF guidelines acknowledge the need for improved assessment of patient-specific hemodynamic measures,^3^ PV loop measurement has historically required invasive measurements of LV pressure, limiting applications.

Recent work has seen the introduction and validation of a fully noninvasive method for computing left ventricular PV loops using cardiovascular magnetic resonance (CMR) imaging and brachial blood pressure.^12^, ^13^, ^14^, ^15^ This method enables the study of unique cardiac functional parameters without the risk, discomfort, and cost associated with invasive measurements. Moreover, as the method only requires time-resolved CMR short-axis images and a concurrent brachial blood pressure recording, it is well suited to retroactively examine long-term outcomes in previously studied HF patients. Whether noninvasive PV loops can add prognostic information in HFrEF is currently unknown.

Here we aimed to investigate whether noninvasively computed PV loops can predict major adverse cardiac events (MACE) in HFrEF patients.

## Methods

### Study sample

The study was approved by the Swedish Ethical Review Authority and the Swedish National Board of Health and Welfare and was conducted in accordance with the Declaration of Helsinki. This was a retrospective analysis of data acquired from patients with clinically diagnosed heart failure who underwent CMR examination at our center between 2004 and 2014. From the records, 287 potential patients with complete CMR data sets, a left ventricular ejection fraction ≤40%, and with written informed consent were identified. Indications for CMR were typically assessment of infarction, fibrosis, ventricular volumes, or investigation prior to implantable cardioverter defibrillator or biventricular pacemaker for resynchronization therapy.

### Data collection

Medical history was obtained from the Cause of Death Registry (Swedish National Board of Health and Welfare). Baseline data were retrospectively obtained on medications, hypertension, diabetes, and NYHA functional classifications through electronic patient records. Patients were classified as either having ischemic or nonischemic cardiomyopathy based on clinical data, history, and the CMR report.

### CMR imaging

All patients had undergone imaging at either 1.5- or 3.0T (both Philips Achieva). The imaging protocols included short- and long-axis (2ch, 3ch, and 4ch) cine images acquired using balanced steady-state free precession imaging, and late gadolinium enhancement (LGE) images in the same views. Sequence-specific parameters are given in the Supplemental Appendix. A brachial blood pressure measurement was performed using a calibrated automatic cuff when resting in the supine position in conjunction with the CMR scan.

### CMR image analysis

Left ventricular endocardial borders were contoured in the short-axis stack over the cardiac cycle using Segment 4.0 R11026 (Medviso), as previously described.^14^^,^^16^ LGE images were visually evaluated for the presence and distribution of scar by the attending physician according to clinical routine and interpreted in light of the clinical data to categorize patients as either ischemic cardiomyopathy (ICM) or nonischemic dilated cardiomyopathy (NIDCM).

PV loops were computed using a previously published^12^ and validated^13^, ^14^, ^15^^,^^17^ model. In summary, the model uses a digitized time-varying elastance function^16^ to estimate dynamic LV pressures in relation to LV volumes from CMR images. The elastance model is scaled in time so that the minimal LV volume (end-systolic time frame) coincides with the middle of the downslope of the elastance function. The elastance curve is also scaled in amplitude to match the LV peak pressure (LVP_systole_, approximated from brachial blood pressure using a previously described expression)^18^ and LV end-diastolic pressure (EDP). EDP is necessarily estimated by the user but within reasonable limits has little effect on the parameters used here;^12^^,^^15^ we set the EDP to 7 mmHg for all data sets.

**Figure 1:**
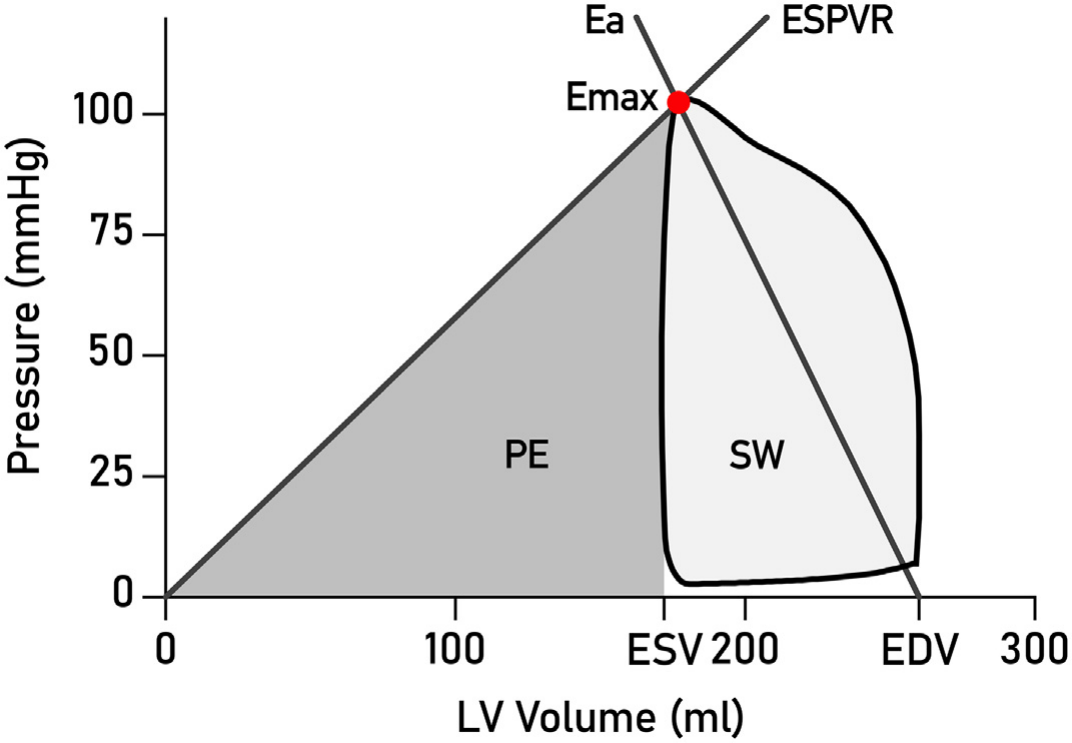
**Left Ventricular PV Loop Computed From CMR Images and Brachial Blood Pressure** The light gray area enclosed by the loop is the stroke work (SW). The dark gray area bounded by the pressure-volume (PV) loop and the end-systolic pressure-volume relationship (ESPVR, Defined as the slope from the origin to the point of maximal elastance emax) is the potential energy (PE). ventricular efficiency (VE) is calculated as SW/(PE + SW). Arterial elastance (Ea) is calculated as the absolute value of the slope from emax to the X axis intercept at end-diastolic volume. CMR = cardiovascular magnetic resonance; EDV = end-diastolic volume; ESV = end-systolic volume.

From the resulting PV loops, we readily obtained the following parameters for evaluation: SW (J), potential energy (PE) (J), ventricular efficiency (VE) (%), mean external power (J/s), contractility (mm Hg/mL), energy per ejected volume (J/mL), and arterial elastance (mm Hg/mL). Figure 1 shows how the different parameters are derived from a PV loop.

### Follow-up

Follow-up data were obtained from the Cause of Death Registry and the Patient Registry (both Swedish National Board of Health and Welfare) and from manual review of electronic patient records for 5 years after CMR. The primary outcome was MACE within the 5-year follow-up period. MACE was defined as cardiovascular death (caused by acute myocardial infarction (AMI), heart failure, or ventricular arrhythmia), or all-cause HF hospitalization, AMI, revascularization (endovascular or coronary bypass graft), cardiac arrest, pulmonary edema, heart transplantation, sustained ventricular tachycardia or ventricular fibrillation, and LV assist device treatment. A complete list of the International Classification of Diseases diagnosis codes used to generate MACE is provided in the Supplemental Appendix. All events, including recurring events such as HF hospitalization were noted, and elapsed time between CMR examination and first MACE was used as input for survival analyses. Patients were censored if they reached the end of the study period (December 31, 2019) without an event, or at a time of noncardiovascular death.

### Statistical analysis

Data distributions were assessed for normality using histograms. Continuous data were tested using Student’s *t*-test or Mann-Whitney *U* test and are presented as mean ± SD or median (IQR) as appropriate. Categorical data were tested using the chi-square test. Statistical significance was assigned at the *P* < 0.05 level.

Time to first event survival analyses were conducted in a stepwise manner using both a-priori based and data-driven variable selections.

First, unadjusted Cox regressions were performed to highlight clinical and imaging parameters of interest. Second, we performed stepwise multivariable Cox regressions. Parameter multicollinearity was assessed using a Spearman correlation matrix, and only pairs of variables with a correlation coefficient <0.8 were allowed in the multivariable regression models. Model 1 (M1) incorporated baseline study sample characteristics: age and sex.

Model 2 (M2) added clinical parameters: M1 + hypertension, diabetes, and etiology of HF. This step was designed to separate patients based on readily available and clinically important mediators for outcome in HF. To preserve statistical power, N-terminal prohormone of brain natriuretic peptide level was not entered in the multivariable model as it was only available in a minority of patients.

Model 2 was subsequently expanded in two different directions. Model 3 *Volumes* (M3_V_) added traditional imaging parameters of LV function: M2 + ejection fraction (EF) and end-diastolic volume index. This model was intended to account for imaging parameters which are generally accepted as prognostically significant, and which are incorporated in the standard clinical phenotyping of heart failure.^3^ Model 3 *Pressure-Volume* (M3_P-V_) added the two PV loop parameters with highest probability of additional value based on the Wald statistic and *P* value: M2 + VE and SW. The M3_V_ and M3_P-V_ models can be understood as branches forking from the M2 model. The added value of each separate model iteration from M1 to M2 and onward to branches M3_V_ and M3_P-V_ was evaluated using the concordance index and the likelihood-ratio test. Event-free survival was visualized using Kaplan-Meier analysis. Statistical analysis was performed in R 4.0.3 (R Core Team) and Prism 10.1.1 (GraphPad Software).

## Results

### Study sample characteristics

After screening data sets for completeness and image quality, 164 patients were included in the final analysis. The median age was 63 years and 79% were male. LGE was present in 78%, and 55% of patients were diagnosed as having ICM. Patients were generally well medicated with cardioprotective treatments available at the time, at the discretion of the treating physician. Patient characteristics including medications are summarized in Table 1.

**Table 1:** Study Sample Baseline Clinical and Cardiovascular Magnetic Resonance Characteristics

	Study Sample (n = 164)	ICM (n = 91)	NIDCM (n = 73)	*P* Value
Age, y	63 (55-70)	66 (59-72)	59 (49-66)	**<0.0001**^a^
Male, %	79%	87%	68%	**0.004**^b^
Body mass index, kg/m^2^	26.2 (24.1-28.7)	26.5 (24.2-28.7)	25.8 (23.9-28.7)	0.452^a^
NYHA functional class, I-IV	I: 16	I: 12	I: 4	0.299^b^
	II: 42	II: 25	II: 17	
	III: 57	III: 28	III: 29	
	IV: 22	IV: 12	IV: 10	
NT-proBNP, μg/L	1,567 (596-3,160)	1,457 (583-2,918)	1,932 (593-3,471)	0.375^a^
Atrial fibrillation, %	23%	25%	19%	0.353^b^
Prior stroke, %	5%	7%	3%	0.255^b^
Prior revascularization, %	41%	66%	10%	**<0.0001**^b^
Hypertension, %	38%	42%	33%	0.244^b^
Diabetes, %	20%	24%	15%	0.148^b^
Smoker, %	15%	20%	10%	0.071^b^
Hyponatremia, %	8%	7%	10%	0.480^b^
eGFR, mL/min/1.73 m^2^	69 ± 19	65 ± 19	74 ± 19	**0.008**
LV EDV index, mL/m^2^	152 (127-178)	148 (129-173)	160 (121-191)	0.432^a^
LV SV index, mL/m^2^	39 ± 10	39 ± 9	38 ± 10	0.861
Cardiac index, L/min	2.69 ± 0.78	2.60 ± 0.76	2.80 ± 0.81	0.107
LGE on CMR, %	78%	97%	55%	**<0.0001**^b^
LV EF, %	26 ± 8	26 ± 8	25 ± 8	0.393
LV AVPD, mm	7.8 ± 2.4	7.7 ± 2.4	7.9 ± 2.5	0.645
GLS, %	7.0 (5.4-9.9)	6.8 (5.4-9.4)	7.4 (5.5-10.4)	0.125
RAAS antagonist, %	88%	84%	93%	0.061^b^
Beta-blocker, %	88%	90%	85%	0.314^b^
Spironolactone, %	49%	44%	56%	0.120^b^
Anticoagulant, %	38%	35%	41%	0.377^b^
Salicylic acid, %	57%	77%	33%	**<0.0001**^b^
Diuretic, %	63%	60%	66%	0.484^b^
Antiarrhythmic drug, %	19%	16%	22%	0.377^b^
Stroke work, J	0.86 (0.63-1.07)	0.89 (0.63-1.07)	0.82 (0.63-1.06)	0.383^a^
Potential energy, J	1.65 (1.26-2.12)	1.65 (1.36-2.03)	1.65 (1.12-2.21)	0.994^a^
Ventricular efficiency, %	34 (27-41)	34 (27-42)	35 (27-41)	0.651
External power, J/s	1.01 (0.79-1.24)	1.01 (0.78-1.23)	1.02 (0.80-1.25)	0.647^a^
Contractility, mm Hg/mL	0.48 (0.36-0.65)	0.49 (0.38-0.65)	0.47 (0.33-0.69)	0.772^a^
Energy per ejected volume, J/mL	0.033 (0.028-0.042)	0.032 (0.028-0.040)	0.034 (0.027-0.042)	0.433^a^
Arterial elastance, mm Hg/mL	1.61 (1.35-1.91)	1.57 (1.35-1.86)	1.68 (1.34-2.09)	0.331^a^

Values are median (IQR) or mean ± SD. *P* values indicated for comparison ICM vs NIDCM. *P* < 0.05 shown in **bold**.

AVPD = atrioventricular plane displacement; CMR = cardiovascular magnetic resonance; EDV = end-diastolic volume; EF = ejection fraction; eGFR = estimated glomerular filtration rate; GLS = global longitudinal strain; ICM = ischemic cardiomyopathy; LGE = late gadolinium enhancement; LV = left ventricular; LVEF = left ventricular ejection fraction; NIDCM = nonischemic dilated cardiomyopathy; NT-proBNP = N-terminal prohormone of brain natriuretic peptide; RAAS = renin-angiotensin-aldosterone system; SV = stroke volume.

a Tested using Mann-Whitney *U*.

b Tested using chi-square test.

### PV loop parameters

**Figure 2:**
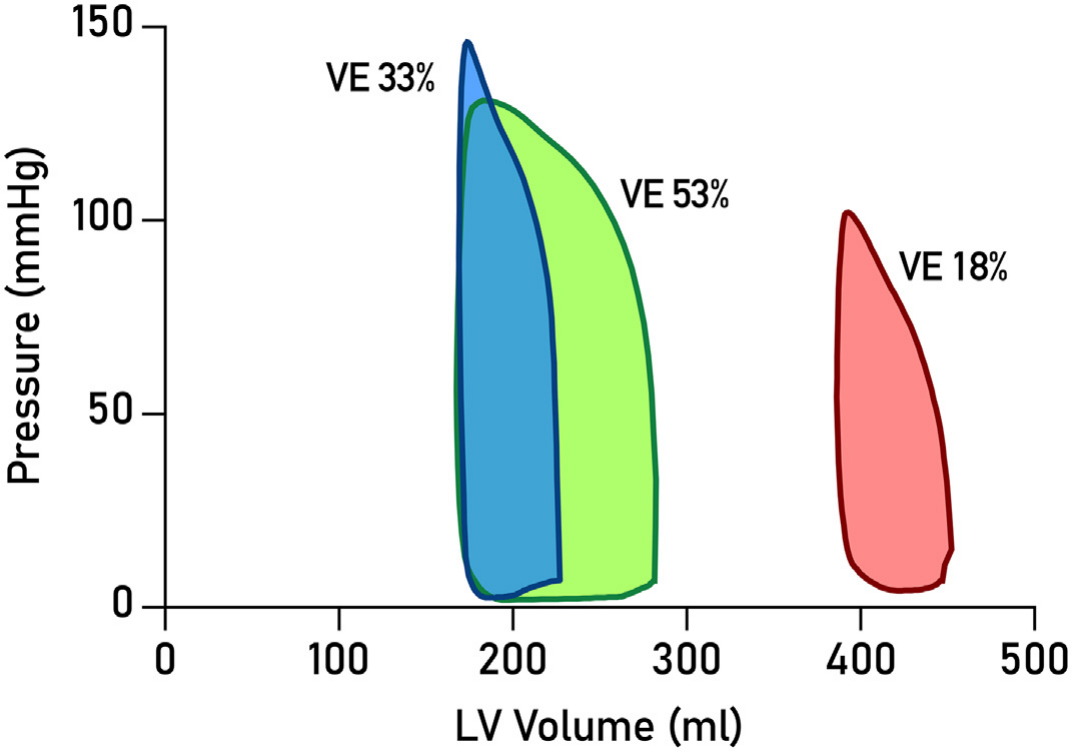
**Noninvasive Pressure-Volume Loops in Heart Failure** Three cases are shown, all with ischemic cardiomyopathy. The green loop illustrates a patient who completed the follow-up with no adverse events. The blue loop shows a patient who was hospitalized due to worsening heart failure after 2.3 years. The red loop is from a patient who died from cardiac arrest 5 weeks after the CMR examination. CMR = cardiovascular magnetic resonance; VE = ventricular efficiency.

Figure 2 shows representative PV loops from three patients, one from each VE tertile. Table 1 shows PV parameters for all patients. Comparison of PV parameters between NIDCM and ICM groups revealed no statistically significant differences (*P* > 0.05 for all).

### Clinical outcomes

Patients were followed for an average of 4.5 years. The primary outcome occurred in 88 individuals after an average of 2.8 years; the most frequently observed was all-cause HF hospitalization (n = 49), followed by cardiovascular death (n = 23), revascularization (n = 8), AMI (n = 6), cardiac arrest (n = 1), and ventricular tachycardia (n = 1).

### Unadjusted and adjusted models

**Figure 3:**
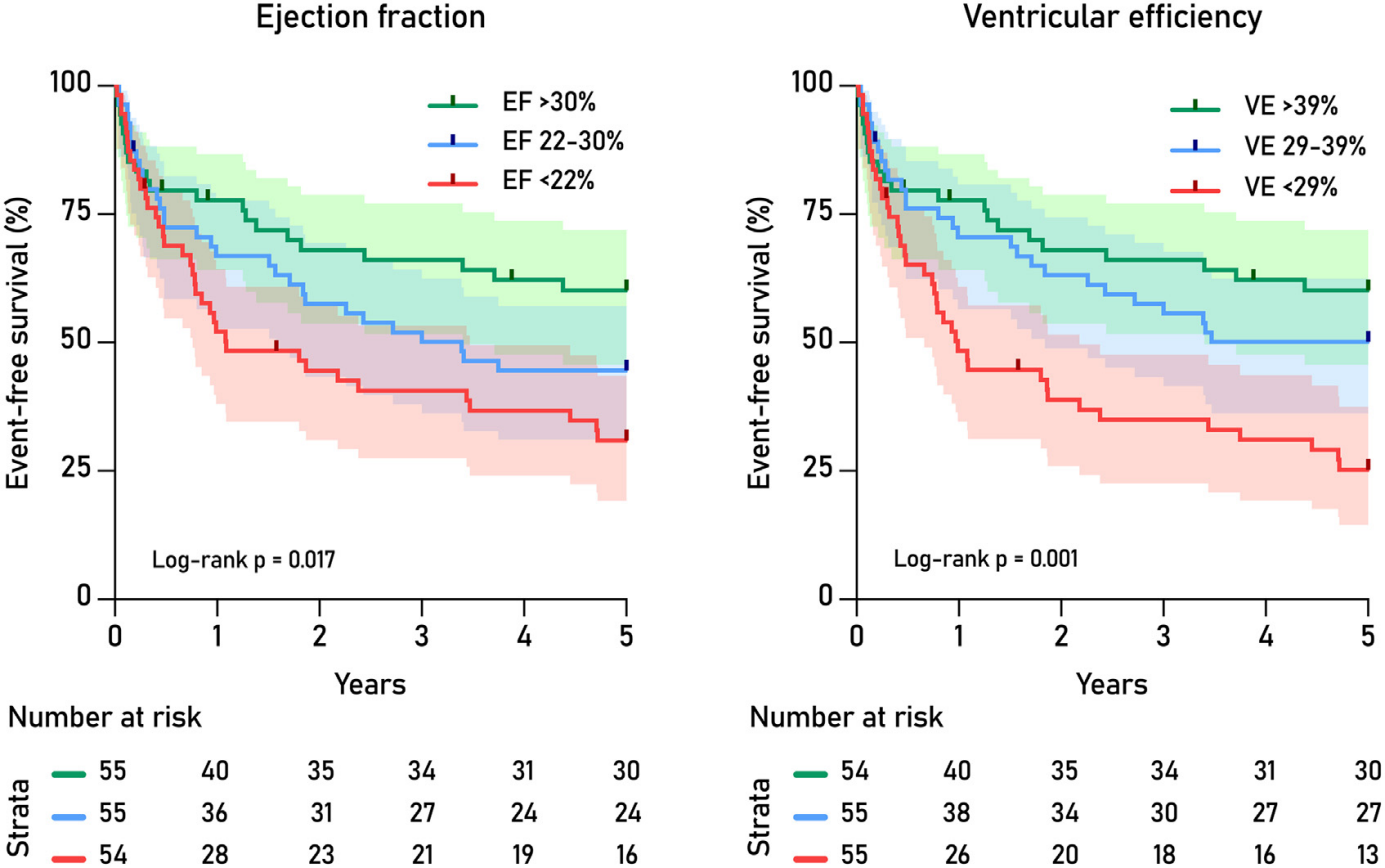
**Kaplan-Meier Plots of Event-Free Survival by Tertiles of Ejection Fraction and Ventricular Efficiency** Shaded areas indicate 95% CIs for the survival function. VE = ventricular efficiency.

**Figure 4:**
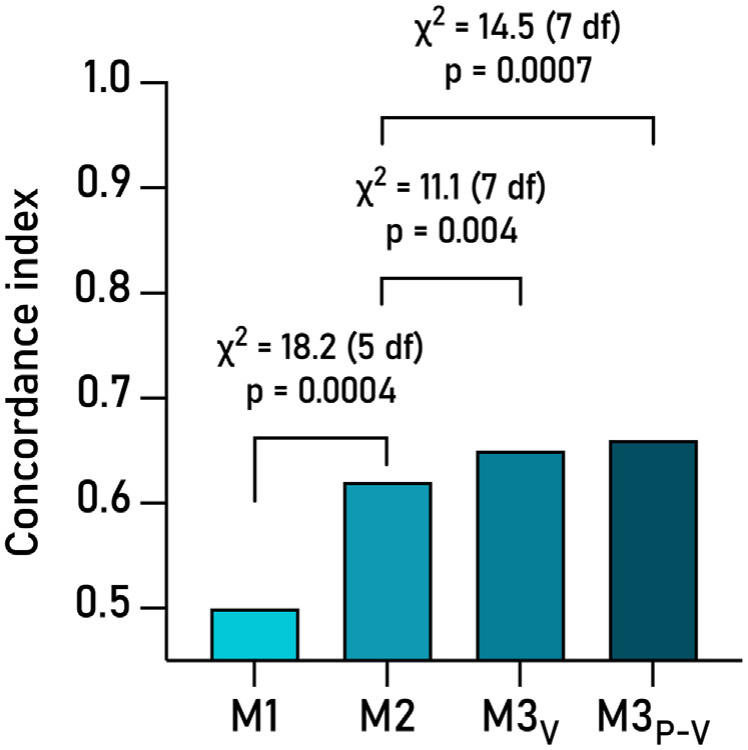
**Concordance and Added Value of Iterative Survival Models M1-M3**_**P-V**_**for Predicting MACE** M1 incorporates baseline demographic data, M2 adds readily available and clinically important mediators for outcome in HF. From there the model was forked to M3_V_, adding traditional volume metrics, and M3_P-V_, instead adding pressure-volume parameters with high wald test statistic from the unadjusted cox regressions. A C-index of 0.5 is equal to coin toss. Chi-square and *P* values for likelihood ratio test. df = degrees of freedom; MACE = major adverse cardiac events.

Unadjusted Cox regression (Table 2) found significant associations with the primary end point for HF etiology, log N-terminal prohormone of brain natriuretic peptide, EF, atrioventricular plane displacement, global longitudinal strain, as well as SW, VE, external power, contractility, and energy per ejected volume. Figure 3 shows Kaplan-Meier plots for EF and VE. In stepwise multivariable Cox regression, the concordance improved significantly for each step from M1 to M3_P-V_ with the likelihood ratio test. In M3_P-V_, only VE and etiology of HF remained significant, with a concordance of 0.66 (Table 3, Figure 4).

**Table 2:** Univariate Cox Regression Model: Association Between Baseline Clinical and Cardiovascular Magnetic Resonance Characteristics and Major Adverse Cardiac Events

	HR (95% CI)	Wald Statistic	*P* Value	n
Presence of LGE	1.55 (0.89-2.70)	2.4	0.122	
Nonischemic heart failure etiology	**0.42** (0.27-0.66)	13.9	**<0.001**	
Male	1.09 (0.66-1.81)	0.1	0.742	
NYHA functional class	1.25 (0.96-1.63)	2.7	0.098	137
Prior stroke	0.97 (0.36-2.65)	0	0.957	
Prior revascularization	1.42 (0.93-2.16)	2.7	0.100	
Atrial fibrillation/flutter	1.28 (0.80-2.06)	1.0	0.308	
Hypertension	0.91 (0.59-1.41)	0.2	0.686	
Diabetes	1.44 (0.88-2.38)	2.1	0.149	
Smoking	1.45 (0.85-2.47)	1.8	0.178	149
Hyponatremia <135 mmol/l	1.90 (0.95-3.79)	3.3	0.069	154
Age, y	1.01 (0.99-1.03)	0.7	0.399	
NT-proBNP, log μg/l	**2.71** (1.54-4.78)	11.9	**0.001**	112
Estimated GFR, mL/min/1.73 m^2^	0.93 (0.82-1.06)	1.1	0.295	151
End-diastolic volume index, 10 mL/m^2^	1.04 (1.00-1.08)	3.0	0.083	
Stroke volume index, 10 mL/m^2^	0.85 (0.69-1.06)	2.2	0.143	
Cardiac index, l/min	0.86 (0.53-1.38)	0.4	0.524	
Left ventricular ejection fraction, %	**0.95** (0.92-0.99)	9.1	**0.003**	
AVPD, mm	**0.88** (0.78-0.96)	7.9	**0.005**	158
Global longitudinal strain, %	**0.88** (0.77-0.94)	13.5	**<0.001**	162
Stroke work, J	**0.42** (0.21-0.81)	6.8	**0.009**	
Potential energy, J	1.16 (0.89-1.52)	1.2	0.276	
Ventricular efficiency, %	**0.96** (0.94-0.98)	12.4	**<0.001**	
External power, J/s	**0.53** (0.29-0.95)	4.6	**0.033**	
Contractility, mm Hg/mL	**0.32** (0.11-0.88)	4.9	**0.027**	
Energy per ejected volume, mJ/mL	**1.02** (1.00-1.04)	5.4	**0.020**	
Arterial elastance, mm Hg/mL	1.38 (0.98-1.96)	3.3	0.068	

*P* < 0.05 are shown in **bold**. n, sample size per analysis for variables available in <100% of subjects.

AVPD = atrioventricular plane displacement; GFR = glomerular filtration rate; LGE = late gadolinium enhancement; NT-proBNP = N-terminal prohormone of brain natriuretic peptide.

**Table 3:** Multivariable Cox Proportional Hazards Models: Association Between Baseline Clinical and Cardiovascular Magnetic Resonance Characteristics and Major Adverse Cardiac Events

	M1		M2		M3_V_		M3_P-V_	
	HR (95% CI)	*P* Value	HR (95% CI)	*P* Value	HR (95% CI)	*P* Value	HR (95% CI)	*P* Value
Age, 1 y	1.00 (0.98-1.02)	0.946	0.98 (0.97-1.00)	0.088	0.99 (0.97-1.01)	0.201	0.99 (0.97-1.01)	0.267
Male	0.98 (0.59-1.62)	0.929	0.71 (0.42-1.21)	0.204	**0.57** (0.33-0.99)	**0.045**	0.58 (0.33-1.02)	0.057
Nonischemic heart failure etiology			**0.36** (0.21-0.60)	**<0.0001**	**0.27** (0.19-0.54)	**<0.0001**	**0.37** (0.20-0.67)	**<0.0001**
Hypertension			0.96 (0.61-1.51)	0.846	1.13 (0.71-1.81)	0.597	1.18 (0.74-1.89)	0.485
Diabetes			1.45 (0.86-2.43)	0.163	1.32 (0.78-2.26)	0.304	1.24 (0.72-2.12)	0.435
Left ventricular ejection fraction, -%					**1.05** (1.01-1.08)	**0.012**		
End-diastolic volume index, 1 mL/m^2^					1.02 (0.96-1.08)	0.574		
Ventricular efficiency, -%							**1.04** (1.01-1.08)	**0.010**
Stroke work, J							0.83 (0.32-2.13)	0.701

Models are for successive model iterations M1 - M3_P-V_.

Model 1 (M1) incorporated baseline study sample characteristics: age and sex.

Model 2 (M2) added clinical parameters: M1 + hypertension, diabetes, and etiology of HF.

Model 3 *Volumes* (M3_V_) added traditional imaging parameters of LV function: M2 + EF and end-diastolic volume index.

Model 3 *Pressure-Volume* (M3_P-V_) added the 2 PV loop parameters with highest probability of additional value based on the Wald statistic and *P* value: M2 + ventricular efficiency and stroke work.

Note ejection fraction and ventricular efficiency are given as negative percentage to associate a decrease with increased HR.

## Discussion

The study demonstrates that VE, derived from noninvasive PV loop analysis of a standard CMR scan at rest, is associated with MACE in heart failure patients with reduced ejection fraction. This is the first study investigating the prognostic value of CMR-derived PV loop parameters in HFrEF patients. In this patient group, PV loop analysis may help improve risk stratification and hence clinical management at minimal expense and with no added risk or discomfort for the patient.

### Clinical implications

The immediate availability of PV loop analysis from routine CMR images and brachial blood pressure, combined with its potential for risk stratification in HFrEF patients, suggests its future implementation in the standard reporting workflow for clinical CMR.^19^ With the development of deep learning-based segmentation algorithms trained on large data sets,^19^^,^^20^ a fully automated in-line assessment of PV loop parameters is feasible. Implementing this workflow on the magnetic resonance imaging reconstruction system would enable online, dynamic assessment of myocardial performance, for example during interventions, as recently suggested by Seemann et al.^21^ Before clinical reporting on myocardial efficiency in HFrEF is implemented, the cutoff values for high/intermediate/low risk require external validation in an independent study.

### Relation to earlier work

The discovery of the end-systolic PV relationship (ESPVR) by Suga et al^22^^,^^23^ was a key development for PV analysis, as the maximal value of the time-varying elastance of the ventricle (E_max_) is a load-independent index of contractility. These findings were later verified in man by Grossman et al^24^ who also demonstrated how V_0_ can be measured from variable ventricular loading. Sagawa et al^25^ introduced the ratio of systolic pressure to end-systolic volume (SP/ESV index, oftentimes using peak SP) as a simplified indicator of ventricular contractility, while later validation suggested its use for clinical studies of heart function.^26^ Subsequent studies found SP/ESV, peak SP/ESV, or ESPVR predictive for long-term outcomes in various patient cohorts.^27^, ^28^, ^29^, ^30^ While we found ESPVR to be significantly associated with MACE in unadjusted Cox regression, other PV loop parameters demonstrated better Wald test and *P* values, and contractility was therefore not entered into the multivariable model. In summary and coherent with our findings, the inability of the failing ventricle to adapt from rest to stress conditions carries prognostic information. The main difference between these studies and ours is that we analyzed rest imaging only.^8^

While our survival model provided good separation between low- and high-risk groups, the addition of stress imaging might further improve its accuracy. Stress CMR may be conducted using dobutamine^31^ which increases contractility, SW, and VE^13^ similarly to physical exercise,^32^ but without the added challenges associated with acquiring CMR images of adequate quality during exercise.^33^, ^34^, ^35^ Dobutamine stress has been shown to unmask energetic deficit across a spectrum of cardiac ailments, including HFrEF.^1^^,^^36^^,^^37^ Similarly, noninvasive PV loop analysis has been employed to study VE and contractility in HFrEF^17^ as well as in AMI.^38^ These studies underscore the value of PV loop analysis in quantifying dynamic changes to ventricular thermodynamic efficiency resulting from either pharmacological intervention or pathophysiological processes.

LGE has been repeatedly shown to predict outcomes in heart failure, including adverse remodeling, morbidity, and mortality in NIDCM,^39^^,^^40^ where an ischemic LGE pattern is associated with worse outcomes.^41^ Most studies on the prognostic value of LGE in DCM have excluded ICM patients.^39^ In our material, LGE was near-universally present in the ICM subgroup which constituted 55% of the study sample, and in more than half of NIDCM patients, which may explain why HF etiology was found a more powerful risk predictor than LGE. More detailed quantification of LGE burden and localization might translate into improved prognostic power, although most studies on LGE in DCM have used either visual assessment or *n*-standard deviations from remote methods, the latter of which is not appropriate for comparisons across different scanners and sequences.^42^

### Methodological considerations

Ejection fraction and VE are geometrically related. As we assumed V_0_ = 0 ml in all cases, the remaining variance arises from the pressure modeling used for SW. The blood pressure function affects both SW (through determining the upper boundary of the PV loop) and PE (through effects on ESPVR, and hence PE area), and a high degree of similarity between the hazard ratios for VE and EF was therefore expected.

**Figure 5:**
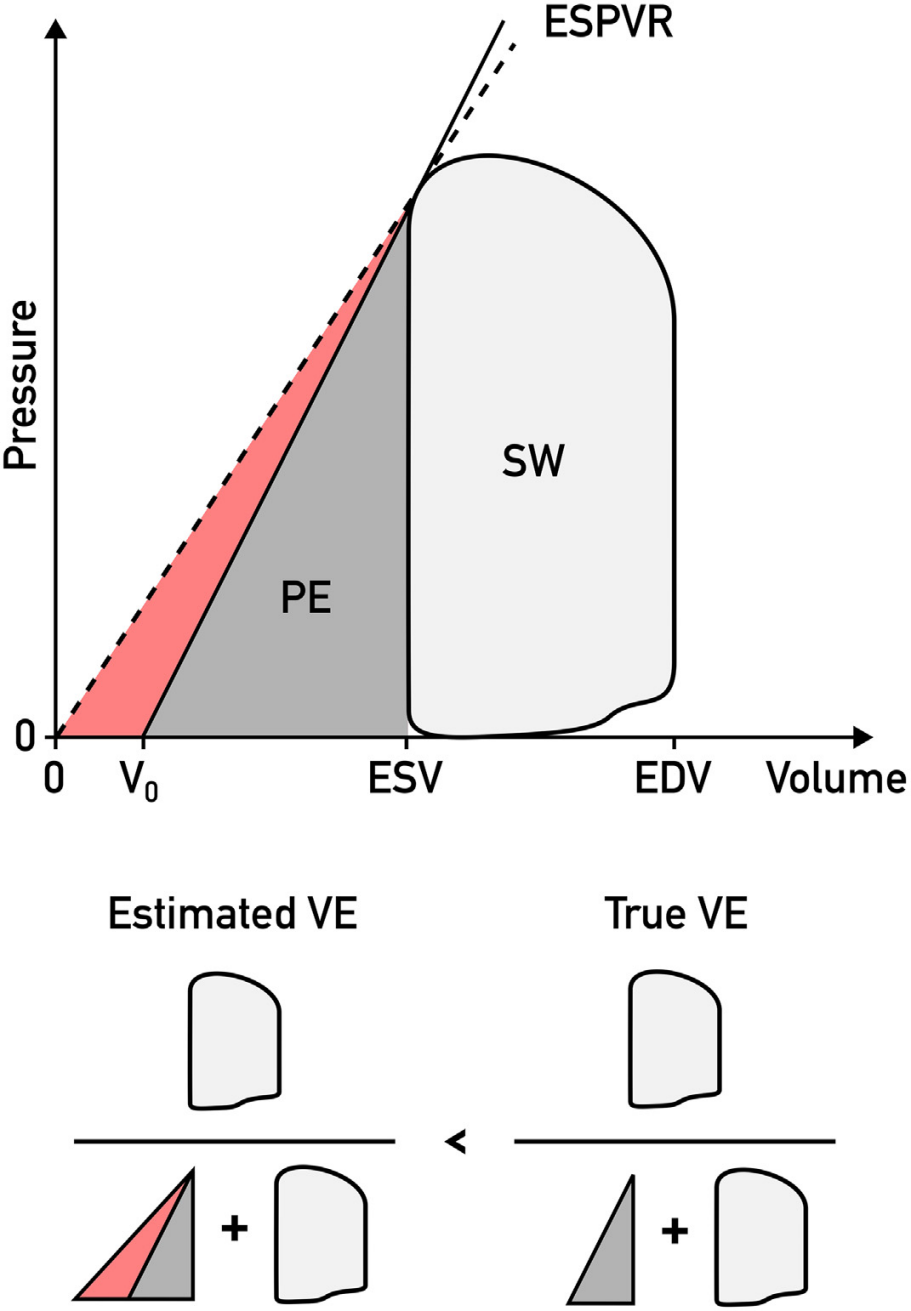
**Effects of Estimated Vs Real V**_**0**_**on Ventricular Efficiency** V_0_ is defined as the (Theoretical) volume at which the ventricle is unloaded. Setting V_0_ to 0 ml, a common simplification, underestimates the end-systolic pressure-volume relationship (ESPVR) (dashed line) and hence overestimates potential energy (PE) (red area), leading to underestimation of ventricular efficiency (VE). EDV = end-diastolic volume; ESV = end-systolic volume; SW = stroke work.

As V_0_ affects PE independently of the PV loop (Figure 5), it is theoretically possible to attain more precise estimates of VE if patient-specific V_0_ can be determined through preload manipulation. As access to data only from one volumetric state was available, we were unable to evaluate the error introduced by setting V_0_ = 0 ml, but it is safe to assume that our VE measurements are underestimated to a variable extent. Better estimates of VE may therefore result in greater distinction from EF and could further improve the predictive model.

To avoid multicollinearity, we opted to evaluate the PV loop metrics separate from the more traditional volumetric variables EF and end-diastolic volume index. We could thus verify both EF and VE as independent predictors of MACE but were unable to directly compare the performance of the two. Despite this, the PV loop model may in fact perform slightly better, as evidenced by the likelihood ratio test. Although EF is more readily computed, VE is a more physiological measure that relates directly to the actual thermodynamic performance of the heart. The well-established predictive power of EF may therefore arise from its close relationship with VE.

### Study Limitations

Standard survival analysis uses time-to-first event as a proxy for the morbidity burden experienced by the individual patient. Recent work has emphasized the added value of analyzing the cumulative disease burden in HF, enabling more comprehensive assessment of the individual case and improved statistical power over traditional time-to-first-event analyses.^43^ The downside is that such statistical models are inherently more complex and may produce unwanted side effects when using composite end points such as MACE. As this was the first attempt at evaluating the predictive value of PV loop analysis, we opted for a simpler survival analysis. Our study sample consisted of HF patients undergoing treatment in the preneprilysin antagonist, pre-sodium/glucose cotransporter 2 inhibitor era. Relatively low event rates indicate patients were generally well managed on therapy available at the time. Follow-up studies on more contemporary patient groups will likely see lower event rates than ours, which may be mitigated using multi-event survival analysis and/or a larger cohort. The predictive accuracy of PV loop analysis may also be affected in the presence of modern drugs such as sodium/glucose cotransporter 2 inhibitor, whose primary mechanism of action in HFrEF remains unclear.^44^ Regardless, we acknowledge that our study was limited in size, and results should be reproduced in a larger cohort before clinical implementation is considered.

Ongoing work using noninvasive PV loop analysis has shown myocardial infarction acutely depresses contractile function in relation to the extent of ischemia (myocardium at risk).^45^ Long-term adaptive remodeling in ICM involves ventricular dilatation and compensatory hypertrophy of viable myocardium. Such changes increase total myocardial metabolic demand and hence the risk for subsequent ischemic events, as well as for worsening heart failure. As our patient material contained a representative mix of etiologies including 55% ischemic patients, we elected to include events related to ischemic heart disease in our definition of MACE.

## Conclusions

VE predicts MACE in patients with HFrEF (Central Illustration). Noninvasive PV loop analysis may add clinical value to a standard CMR scan without incurring additional costs or risk to the patient.

PERSPECTIVES **COMPETENCY IN MEDICAL KNOWLEDGE:** This article demonstrates the association between left ventricular PV loops and MACE in HFrEF. In this patient group, contractile performance and hence ventricular thermodynamic efficiency is impaired to a varying extent. PV loop analysis combines the two most central aspects of cardiac function to enable analysis of parameters which are unavailable from standard volumetric assessment. The results suggest PV loop analysis may offer added value for individualized prognostication compared to base volumetry. Furthermore, the well-established prognostic value of ejection fraction may be explained by its close relationship with VE. Assuming a thermodynamics-oriented mindset in the clinic enables a more sophisticated understanding of cardiac performance and function under different pathophysiological conditions.

**TRANSLATIONAL OUTLOOK:** This study is the first exploration of the predictive value of noninvasive PV loops from CMR in a clinical cohort of heart failure patients. The methods used herein involve standard time-resolved cine imaging and a brachial blood pressure acquired in conjunction with the CMR exam. The analytical framework requires time-resolved delineation of the left ventricular endocardial boundary, either from manual delineation (time and labor intensive) or from automatic or semiautomatic contouring algorithms, whose performance is rapidly approaching that of manual delineations performed by expert readers in core CMR laboratories. Noninvasive PV loop analysis is therefore already available for incorporation in the clinical routine. Future studies should seek to further evaluate the prognostic value of PV loop analysis across different pathologies and in larger cohorts.

**Central Illustration:**
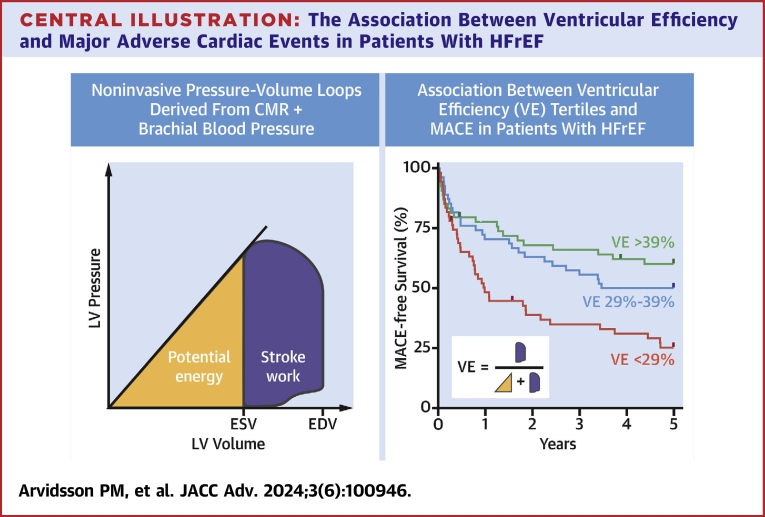
**The Association Between Ventricular Efficiency and Major Adverse Cardiac Events in Patients With HFrEF** Noninvasive pressure-volume loops (thick border) may be computed from CMR and brachial blood pressure. This analysis framework enables calculation of stroke work (purple area) and potential energy (yellow area) and hence ventricular efficiency (VE), which predicts 5-year occurrence of major adverse cardiac events in patients with heart failure with reduced ejection fraction (HFrEF). CMR = cardiovascular magnetic resonance; EDV = end-diastolic volume; ESV = end-systolic volume; MACE = major adverse cardiac events.

## Funding support and author disclosures

Dr Arvidsson is supported by Swedish governmental funding of clinical research (ALF), the Swedish Heart and Lung Foundation (grant no 20210635), the Region of Scania, Sweden, Bundy Academy, and 10.13039/501100008310Lisa och Johan Grönbergs Stiftelse. Dr Arheden is supported by Swedish governmental funding of clinical research (ALF), the Swedish Heart and Lung Foundation (grant no 2020030322), and the Region of Scania, Sweden. All other authors have reported that they have no relationships relevant to the contents of this paper to disclose.
