# Supplementary material for: Noninvasive Pressure-Volume Loops Predict Major Adverse Cardiac Events in Heart Failure With Reduced Ejection Fraction
Source: JACC Adv. 2024 May 4;3(6):100946. doi: 10.1016/j.jacadv.2024.100946 (PMC11198266; doi:10.1016/j.jacadv.2024.100946)
Supplement: Supplemental Data [file mmc1.docx]

**SUPPLEMENTAL APPENDIX**

**Supplemental methods**

**CMR Protocol**

Typical imaging parameters for bSSFP were: echo time (TE)/repetition time (TR) 1.6/3.2 ms and flip angle 60° (1.5T), or TE/TR 1.8/3.7 ms and flip angle 45° (3T). Matrix size was typically 256x256 with acquired spatial resolution 1.48x1.48x8 mm and no slice gap. Retrospective ECG gating was used with 30 reconstructed time phases, typically resulting in a reconstructed temporal resolution of around 35 ms.

For late gadolinium enhancement (LGE), we used either a 2D phase-sensitive inversion recovery (PSIR) or a 3D IR sequence with matrix size 256x256 and acquired spatial resolution 1.36x1.36x8 mm for both sequences. Imaging parameters for 2D PSIR were typically: TE 4 ms, effective TR every second heartbeat, and flip angle 25°; and for 3D IR, TE 1.3 ms, effective TR every heartbeat, and flip angle 15°. The inversion time was chosen to provide optimal nulling of remote myocardium, typically 280-350 ms.

**List of ICD-10 identifier codes used for generating MACE**

| Major adverse cardiovascular events | ICD-10 codes |
| --- | --- |
| Cardiovascular death | - |
| AMI | I21-I24 |
| Revascularization | FNG, FNA, FNC |
| Heart failure hospitalization | I25, I420, I50 |
| Heart transplant | FQA, FQB |
| Cardiac arrest | I46 |
| Ventricular arrythmia | I472B, I472C, I490 |
| Lung edema | J81 |
| Ventricular Assist device | FXL, FXN |
